# Supplementary material for: How well does the virtual format of oncology multidisciplinary team meetings work? An assessment of participants’ perspectives and limitations: A scoping review
Source: PLoS One. 2023 Nov 16;18(11):e0294635. doi: 10.1371/journal.pone.0294635 (PMC10653537; doi:10.1371/journal.pone.0294635)
Supplement: S2 File — This file contains the individual search strings used for each database to perform the literature search for this study. (PDF) [file pone.0294635.s002.pdf]

Supplementary File 2. Search strings used for database searches.

MEDLINE

((cancer boards[Title/Abstract]) OR (Cancer conferences[Title/Abstract]) OR (cancer meetings[Title/Abstract]) OR (cancer multidisciplinary teams[Title/Abstract]) OR (case conferences[Title/Abstract]) OR (case discussions[Title/Abstract]) OR (case presentations[Title/Abstract]) OR (case reviews[Title/Abstract]) OR (clinical conferences[Title/Abstract]) OR (clinical meetings[Title/Abstract]) OR (clinical teams[Title/Abstract]) OR (interdisciplinary conferences[Title/Abstract]) OR (interdisciplinary meetings[Title/Abstract]) OR (interdisciplinary teams[Title/Abstract]) OR (multidisciplinary cancer care[Title/Abstract]) OR (multidisciplinary cancer conferences[Title/Abstract]) OR (multidisciplinary cancer teams[Title/Abstract]) OR (multidisciplinary clinics[Title/Abstract]) OR (multidisciplinary communication[Title/Abstract]) OR (multidisciplinary conferences[Title/Abstract]) OR (multidisciplinary consultations[Title/Abstract]) OR (multidisciplinary discussions[Title/Abstract]) OR (multidisciplinary meetings[Title/Abstract]) OR (multidisciplinary reviews[Title/Abstract]) OR (multidisciplinary teams[Title/Abstract]) OR (multidisciplinary team management[Title/Abstract]) OR (multidisciplinary team meetings[Title/Abstract]) OR (Multidisciplinary teamwork[Title/Abstract]) OR (multidisciplinary tumor boards[Title/Abstract]) OR (multidisciplinary tumour boards[Title/Abstract]) OR (multidisciplinary tumour board meetings[Title/Abstract]) OR (oncology boards[Title/Abstract]) OR (oncology conferences[Title/Abstract]) OR (oncology meetings[Title/Abstract]) OR (tumor boards[Title/Abstract]) OR (tumor conferences[Title/Abstract]) OR (tumor meetings[Title/Abstract]) OR (tumour boards[Title/Abstract]) OR (tumour conferences[Title/Abstract]) OR (tumour meetings[Title/Abstract]))) OR (Patient care team[MeSH Terms])) OR (interdisciplinary studies[MeSH Terms])) OR (interdisciplinary communication[MeSH Terms]))

AND

(neoplasm[MeSH Terms]) OR (cancer) OR (oncology)

AND

(Virtual OR online OR Virtual Medicine OR telemedicine OR telehealth OR viruality)

<u>Filters</u>

Time limit: None
Language: English

Embase

(((‘cancer board’/exp) OR (‘cancer conference’/exp) OR (‘cancer meeting’/exp) OR (‘cancer multidisciplinary team’’/exp) OR (‘case conference’/exp) OR (‘case discussion’/exp) OR (‘case presentation’/exp) OR (‘case review’/exp) OR (‘clinical conference’/exp) OR (‘clinical meeting’/exp) OR (‘clinical team’/exp) OR (‘interdisciplinary conference’/exp) OR (‘interdisciplinary meeting’/exp) OR (‘interdisciplinary team’/exp) OR (‘multidisciplinary cancer care’/exp) OR (‘multidisciplinary cancer conference’/exp) OR (‘multidisciplinary cancer team’/exp) OR (‘multidisciplinary clinic’/exp) OR (‘multidisciplinary communication’/exp) OR (‘multidisciplinary conference’/exp) OR (‘multidisciplinary consultation’/exp) OR (‘multidisciplinary discussion’/exp) OR (‘multidisciplinary meeting’/exp) OR (‘multidisciplinary review’/exp) OR (‘multidisciplinary team’/exp) OR (‘multidisciplinary team management’/exp) OR (‘multidisciplinary team meeting’/exp) OR (‘multidisciplinary teamwork’/exp) OR (‘multidisciplinary tumor board’/exp) OR (‘multidisciplinary tumour board’/exp) OR (‘multidisciplinary tumour board meeting’/exp) OR (‘oncology board’/exp) OR (‘oncology conference’/exp) OR (‘oncology meeting’/exp) OR (‘tumor board’/exp) OR (tumor conferenc*) OR (‘tumor meeting’/exp) OR (‘tumour board’/exp) OR (tumour conference*) OR (’tumour meeting’/exp) OR (‘patient care team’/exp) OR (‘interdisciplinary studies’/exp) OR (‘interdisciplinary communication’/exp)):ab,ti)

AND

(((‘neoplasm’/exp) OR (‘cancer’/exp) OR (‘oncology’/exp)):ab,ti)

AND

(((virtual* OR ‘online’/exp OR virtual Medicine OR telemedicine OR telehealth)):ab,ti)

Filters
Time limit: None
Language: English

CINAHL

(((cancer board*) OR (Cancer conference*) OR (cancer meeting*) OR (cancer multidisciplinary team*) OR (case conference*) OR (case discussion*) OR (case presentation*) OR (case review*) OR (clinical conference*) OR (clinical meeting*) OR (clinical team*) OR (interdisciplinary conference*) OR (interdisciplinary meeting*) OR (interdisciplinary team*) OR (multidisciplinary cancer care) OR (multidisciplinary cancer conference*) OR (multidisciplinary cancer team*) OR (multidisciplinary clinic*) OR (multidisciplinary communication*) OR (multidisciplinary conference*) OR (multidisciplinary consultation*) OR (multidisciplinary discussion*) OR (multidisciplinary meeting*) OR (multidisciplinary review*) OR (multidisciplinary team*) OR (multidisciplinary team management) OR (multidisciplinary team meeting*) OR (Multidisciplinary teamwork) OR (multidisciplinary tumor board*) OR (multidisciplinary tumour board*) OR (multidisciplinary tumour board meeting*) OR (oncology board*) OR (oncology conference*) OR (oncology meeting*) OR (tumor board*) OR (tumor conferenc*) OR (tumor meeting*) OR (tumour board*) OR (tumour conference*) OR (tumour meeting*) OR (Patient care team*) OR (interdisciplinary studie*) OR (interdisciplinary communication*)):ab,ti)

AND

(((neoplasm*) OR (cancer*) OR (oncolo*)):ab,ti)

AND

(((Virtual* OR online OR virtual Medicine OR telemedicine OR telehealth)):ab,ti)

Filters
Time limit: None
Language: English

Google Scholar

(Virtual tumor boards)

Filters
Time limit: None
Language: English
