# Supplementary material for: How well does the virtual format of oncology multidisciplinary team meetings work? An assessment of participants’ perspectives and limitations: A scoping review
Source: PLoS One. 2023 Nov 16;18(11):e0294635. doi: 10.1371/journal.pone.0294635 (PMC10653537; doi:10.1371/journal.pone.0294635)
Supplement: S3 File — This file contains the list of studies that were excluded, along with their reasons for exclusion, after full-text review. (PDF) [file pone.0294635.s003.pdf]

**Supplementary File 3**. List of studies that were excluded during full-text review.

| Year | Author | Reason for exclusion |
|---|---|---|
| 2023 | Valerio MR *et al.* | Not VMDTM Perspective |
| 2023 | Oxley S *et al.* | Not VMDTM |
| 2022 | Davis CH *et al.* | Not VMDTM Perspective |
| 2022 | Irwin KE *et al.* | Not VMDTM Perspective |
| 2022 | Chen M *et al.* | Not VMDTM |
| 2022 | Cives M *et al.* | Not VMDTM |
| 2022 | Thompson JA *et al.* | Not VMDTM |
| 2022 | Hellingman T *et al.* | Not VMDTM |
| 2022 | Karzai F *et al.* | Not VMDTM |
| 2022 | Javed AA *et al.* | Not VMDTM |
| 2022 | Brims FJH *et al.* | Not VMDTM |
| 2022 | Segelov E *et al.* | Case Report |
| 2022 | Hopkins SE *et al.* | Could not be retrieved |
| 2021 | Alanie OM *et al.* | Not VMDTM Perspective |
| 2021 | Martínez-Hernández NJ *et al.* | Not VMDTM Perspective |
| 2021 | Pomej K *et al.* | Not VMDTM |
| 2021 | Gatellier L *et al.* | Not VMDTM |
| 2021 | Weickert MO *et al.* | Not VMDTM |
| 2021 | Hasson SP *et al.* | Not VMDTM |
| 2021 | Schäfer N *et al.* | Not Exclusively VMDTM |
| 2021 | Henderson F Jr *et al.* | Case Report |
| 2021 | van Huizen LS *et al.* | Review |
| 2020 | Salari A *et al.* | Not VMDTM Perspective |
| 2020 | Elkaddoum R *et al.* | Not VMDTM Perspective |
| 2020 | Tashkandi E *et al.* | Not VMDTM |
| 2020 | Depypere LP *et al.* | Not VMDTM |
| 2020 | Turri-Zanoni M *et al.* | Not VMDTM |
| 2020 | Rajasekaran RB *et al.* | Not VMDTM |
| 2020 | Ambrosini F *et al.* | Not VMDTM |
| 2020 | Nilsen ML *et al.* | Not VMDTM |
| 2019 | Díez JJ *et al.* | Not VMDTM |
| 2019 | Zhu X *et al.* | Not VMDTM |
| 2019 | Takeda T *et al.* | Not VMDTM Perspective |
| 2018 | Aston SJ *et al.* | Not Oncological VMDTM |
| 2018 | Mori S *et al.* | Not Exclusively VMDTM |
| 2017 | Pedrosa F *et al.* | Not VMDTM Perspective |
| 2017 | Snyder J *et al.* | Not VMDTM |
| 2015 | Salami AC *et al.* | Not VMDTM Perspective |
| 2013 | Seeber A *et al.* | Not VMDTM Perspective |
| 2012 | Stevens G *et al.* | Not VMDTM Perspective |
| 2012 | Xylinas E *et al.* | Case Report |
| 2008 | Dickson-Witmer D *et al.* | Not VMDTM Perspective |
| 2007 | Qaddoumi I *et al.* | Not VMDTM |
| 2005 | Lutterbach J *et al.* | Not VMDTM |
| 2005 | Bauman G *et al.* | Could not be retrieved |
| 2005 | Stalfors J *et al.* | Not VMDTM Perspective |
| 2005 | Fielding RG *et al.* | About future use of VMDTMs |
| 2005 | Kunkler IH *et al.* | Not VMDTM Perspective |
| 2004 | Davison AG *et al.* | Not VMDTM Perspective |
| 2003 | Coleman RL | Abstract |
| 2003 | Barry N *et al.* | Not VMDTM Perspective |
| 2003 | Stalfors J *et al.* | Not VMDTM |
| 2002 | Billingsley KG *et al.* | Not VMDTM Perspective |
| 2002 | Axford AT *et al.* | Not VMDTM Perspective |
| 2001 | Stalfors J *et al.* | Not VMDTM |

| | | |
|---|---|---|
| 2001 | Mizushima H *et al.* | Not VMDTM |
| 2000 | Atlas I *et al.* | Case Report |
| 2000 | Schwieler A *et al.* | Opinion |
| 1998 | Doolittle GC *et al.* | Not VMDTM |
| 1998 | Kunkler IH *et al.* | Not VMDTM |
| 1998 | Stitt JA. | Not VMDTM |
| 1997 | Dolittle GC, Williams A, *et al.* | Not VMDTM |
| 1997 | Doolittle GC, Allen A. | Not VMDTM |
| 1995 | Allen A *et al.* | Not VMDTM |
