# Supplementary material for: How well does the virtual format of oncology multidisciplinary team meetings work? An assessment of participants’ perspectives and limitations: A scoping review
Source: PLoS One. 2023 Nov 16;18(11):e0294635. doi: 10.1371/journal.pone.0294635 (PMC10653537; doi:10.1371/journal.pone.0294635)
Supplement: S4 File — This file highlights further characteristics of included studies, their populations, and VMDTM composition. (PDF) [file pone.0294635.s004.pdf]

Supplementary File 4. Further characteristics of included studies, their populations, and VMDTM composition.

Study	Type of VMDTM	Participant characteristics	Composition of VMDTM and percentage of responses
Ali SR *et al.* 2023	Skin	*Level of training*: completed, 80.9%; registrar/fellow, 8.8%; other, 10.3%.	Surgeon, 21 (30.9%); Dermatologist, 16 (23.5%); Nurse, 15 (22.1%); Oncologist, 5 (7.4%); Radiologist, 4 (5.9%); Coordinator/administrative, 3 (4.4%); Pathologist, 3 (4.4%); GP with an Extended Role in Dermatology, 1 (1.5%).
Amin NB *et al.* 2023	Head and neck	*Years in practice*: <1 year, 1 (2.0%); 1-5 years, 10 (20.0%); 6-10 years, 9 (18.0%); 11-20 years, 13 (26.0%); >20 years, 17 (34.0%).	Surgeon, 11 (22.0%); Radiation oncologist, 19 (38.0%); Medical oncologist, 8 (16.0%); Pathologist, 2 (4.0%); Neuroradiologist, 2 (4.0%); Other, 8 (16.0%).
Groothuizen JE *et al.* 2023	Lung	NR	*Interviews*: Respiratory physicians, 9; Thoracic surgeons, 3; Oncologists, 3; Radiologists, 7; Pathologists, 3; Clinical Nurse Specialists, 4; Multidisciplinary team coordinators, 7; Other clinical professionals, 0; Other non-clinical (e.g. managers), 5.<br><br>*Survey*: Respiratory physicians, 19; Thoracic surgeons, 4; Oncologists, 7; Radiologists, 12; Pathologists, 5; Clinical Nurse Specialists, 12; Multidisciplinary team coordinators, 7; Other clinical professionals, 3; Other non-clinical (e.g. managers), 4.
Soukup T *et al.* 2023	Many	Average years of work experience in these roles was 7 (range, 1-10).<br><br>*Years in practice*:<br>1-2 years, 82; 3-7 years; 118; >8 years, 202.	Allied Health Professionals, 10; Cancer Nurse Specialists, 45; Coordinating, Administrative and Management, 105; Oncologists, 67; Pathologist, 7; Physician, 36; Radiologist, 12; Surgeons, 135.
Thiagarajan S *et al.* 2023	Head and Neck	NR	NR
Bonanno N *et al.* 2023	Many	*Weekly attendance at VMDTMs*: 1-3, 129 (63.2%); 3-6, 18 (8.8%); did not attend every week, 57 (27.9%).<br><br>*Participated in VMDTMs prior to the pandemic*: yes, 74 (36.3%).	Departmental Chairs/Directors, 28 (13.7%); Radiology Consultants, 63 (30.9%); Radiology Fellows/ Residents, 24 (11.8%); Non-Consultant Board Certified Radiologists, 89 (43.6%).
Esteso F *et al.* 2022	Gastrointestinal	NR	NR
Pearlmutter B *et al.* 2022	Many	*Level of training*: completed training, 228 (90.1%); trainee, 25 (9.9%).<br><br>*Years in practice (since training)**: 0-5, 55 (24.1%); 6-10, 53 (23.2%); 11-15, 33 (14.5%); 16+, 86 (37.7%).<br><br>*VMDTMs attended per week*: one, 169 (66.8%); two, 66 (26.1%); three, 15 (5.9%); four or more, 3 (1.2%).<br><br>*One response unknown	Nurses/Advanced Practice Provider, 62 (24.5%); Surgeon, 48 (19.0%); Radiology and Interventional Radiology, 42 (16.6%); Medical and Radiation Oncology, 38 (15%); Pathologist, 26 (10.3%); Others, 17 (6.7%); Medical Specialists, 15 (5.9%); Other Specialists, 5 (2.0%)
Thallinger C *et al.* 2022	Many	NR	NR
Cathcart P *et al.* *2021*	Breast	NR	NR
Mohamedbhai H *et al.* 2021	Head and neck	*Level of training*: completed training, 71; registrar/resident, 16; junior trainee/intern, 13.	Coordinator/administrative, 4 (4.1); Dietician, 9 (9.3); Nurse, 12 (12.4); Oncologist, 5 (5.1); Other, 10 (10.3); Pathologist, 2 (2.1); Radiologist, 8 (8.3); Speech and language therapist, 5 (5.1); Surgeon, 42 (43.3).
Rajasekaran RB *et al.* 2021	Musculoskeletal	Previously participated in VMDTMs prior to the pandemic, 20 (55.6%); Participated only in sarcoma MDT. 17 (47.2%).	Orthopedic Surgeons (consultants and fellows), 22.2% (8/36); Administrative Support Staff, 16.6% (6/36); Radiologists,13.8% (5/36); Plastic Surgeons, 8.3% (3/36); Clinical Oncologists (medical oncology and radiation oncology), 8.3% (3/36); Sarcoma Specialist Nurses, 8.3% (3/36); Pathologists, 5.6% (2/36); Spine Surgeons, 5.6% (2/36); Pediatric Orthopedic Surgeons, 5.6% (2/36); Cardiothoracic Surgeons, 5.6% (2/36).
Rosabal-Obando M *et al.* 2021	Pediatric Central Nervous System	NR	NR
Dharmarajan H *et al.* 2020	Head and neck	*Number of virtual MDC attended*: One, 3 (15.8); Two. 7 (36.8); Three, 9 (47.4).	Otolaryngologist, 9 (47.4); Medical Oncologist, 2 (10.5); Radiation Oncologist, 3 (15.8); Neuroradiologist, 4 (21.1); Pathologist, 1 (5.3); Palliative and Supportive Care, 0 (0).<br><br>Graduate Medical Trainee: No, 15 (78.9); Yes, 4 (21.1).
Habermann TM *et al.* 2020	Lymphoma	NR	NR
Pan M. et al 2020	Sarcoma	NR	Medical oncologists, Radiation oncologists, Pathologists, General Surgeons, Surgical Oncologists, Musculoskeletal oncologists, Pediatric oncologists, Radiologists.
Rosell L *et al.* 2020	Penile, Anal, Vulvar, Childhood, Advanced Esophageal and Hepatobiliary	NR	Surgery, 53%; medicine/oncology, 26%; radiology, 6%; pathology, 2%; none of the above, 14%..<br><br>Physicians, 87%; nurses, 11%; medical secretaries, 2%.
Sidpra J *et al.* 2020	NR	NR	Clinicians, 46%; radiologists, 54%.
Rosell L *et al.* 2019	Penile, Anal, Vulvar, Childhood, Advanced Esophageal and Hepatobiliary		Surgery, 56%; medicine/oncology, 26%; pediatric oncology, 10%; radiology, 6%; pathology, 2%.<br><br>Physicians, 87%; nurses, 11%; medical secretaries, 2%.
van Huizen LS *et al.*	Head and neck	NR	Interviews: oral and maxillofacial surgery, 2; ear, nose and throat, 2;

2019			radiotherapy, 2.
Abu Arja MH *et al.* 2018	Central Nervous System	*Attendance in meetings*: frequent-attendance, 61; weekly, 21; bi-weekly, 25; once a month, 15; infrequent-attendance, 23; once in 3 months, 4; once or twice only, 19; never participated, 11.	NR
Crispen C *et al.* 2014	Breast, Cervical, Head and Neck, Prostate	NR	Radiation Oncologists, 4; Medical Physicists, 4; Medical Dosimetrists, 2.
Marshall CL *et al.* 2014	Many	*Years in practice*: <5 years, 9 (25%); 5–10 years, 9 (25%); 11–15 years, 0 (0%); 16–20 years, 5 (14%); >20 years, 13 (36%)	Medical Oncology, 8 (22%); Radiation Oncology, 5 (14%); General Surgery, 1 (3%); Surgical Oncology ± Hepatobiliary, 5 (14%); Gastroenterology, 1 (3%); Interventional Radiology, 1 (3%); Radiology, 3 (8%); Pathology, 2 (6%); Nuclear Medicine, 1 (3%); Internal Medicine/Pulmonary, 1 (3%); Cancer Registrar, 4 (11%); Other 4 (11%)
Shea CM *et al.* 2014	Breast, Gastrointestinal, Head and Neck, Malignant Hematology	NR	NR
Bold RJ *et al.* 2013	Breast, Genitourinary, Thoracic	NR	Anesthesia,1; Clinical Trials, 1; Gastrointestinal, 5; Interventional Radiology, 9; Medical Student, 2; Medical Oncology, 168; Nuclear Medicine, 13; Pathology, 47; Pulmonary, 1; Radiation Oncology, 46; Radiology, 37; Registered Nurse, 2; Surgery, 25; No Response, 41.
Stevenson MM *et al.* 2013	Lung	NR	Physicians, 6; Nurse Practitioner, 1; Research Staff, 1; Administrative Staff, 1; Tumor Registry Staff, 1.
Donnem T *et al.* 2012	Many	NR	NR
Schroeder JK *et al.* 2011	Breast, Gynecology		
Chekerov R *et al.* 2008	Gynecology	NR	NR
Kunkler IH *et al.* 2007	Breast	NR	NR
Savage SA *et al.* 2006	Head and neck	NR	NR
Kunkler I *et al.* 2006	Breast	NR	NR
Delaney G *et al.* 2004	Breast	NR	NR
Gagliardi *et al.* 2003	Breast, Colorectal, Gynecology, Lung	NR	NR
Oliver IN *et al.* 2000	Breast and others	NR	Medical oncologist, 7; Radiation oncologist, 3; Nurse, 3; General physician, 2; Palliative care clinician, 2; General surgeon, 1; Pathologist, 1; Radiologist, 1.
Hunter et al. 1999	Many	NR	NR
	Breast	NR	NR

Abbreviations: NR, not reported; VMDTM, virtual multidisciplinary team meeting.

Note: The numbers quoted in the table are of respondents who completed surveys and whose data was incorporated into the included study’s analysis.
