# Supplementary material for: How well does the virtual format of oncology multidisciplinary team meetings work? An assessment of participants’ perspectives and limitations: A scoping review
Source: PLoS One. 2023 Nov 16;18(11):e0294635. doi: 10.1371/journal.pone.0294635 (PMC10653537; doi:10.1371/journal.pone.0294635)
Supplement: S5 File — This file shows the country/region-wise distribution of populations in included studies. (PDF) [file pone.0294635.s005.pdf]

Supplementary File 5. Country/region-wise distribution of populations in included studies.

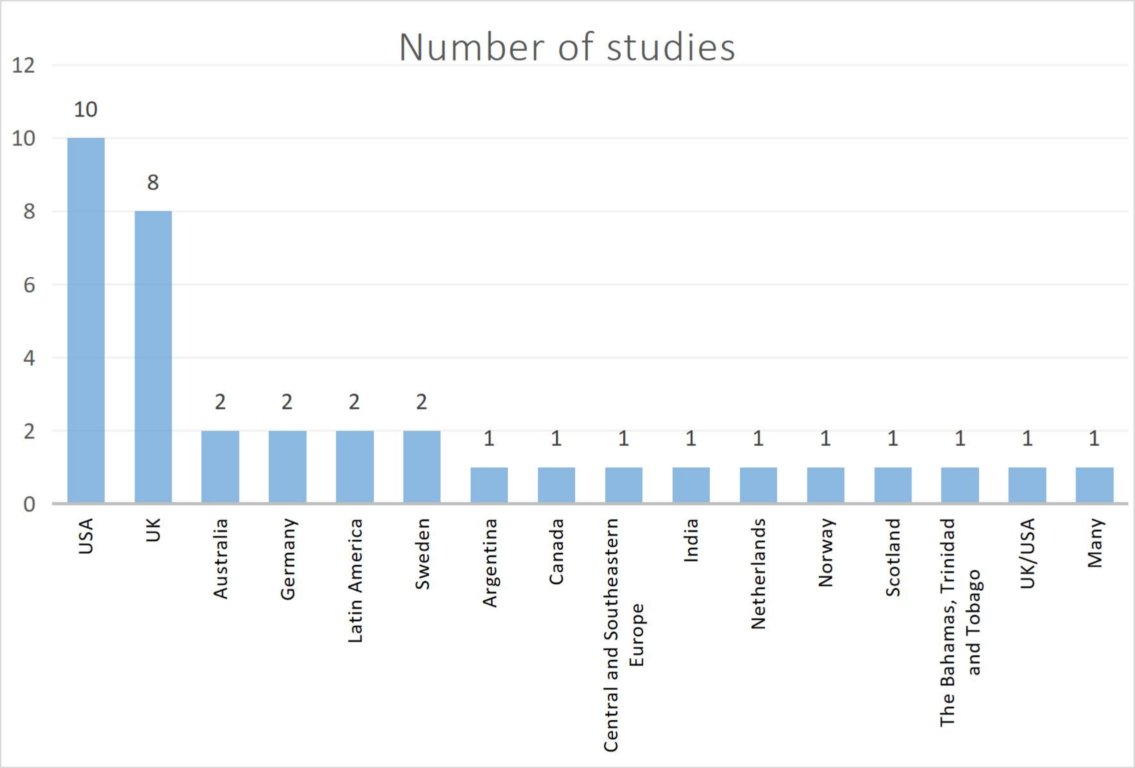

Abbreviations: UK, United Kingdom; USA, United States of America.

The figure describes the geographical distribution of populations/survey respondents in each study that was included in this review (n=36).
