# Supplementary material for: How well does the virtual format of oncology multidisciplinary team meetings work? An assessment of participants’ perspectives and limitations: A scoping review
Source: PLoS One. 2023 Nov 16;18(11):e0294635. doi: 10.1371/journal.pone.0294635 (PMC10653537; doi:10.1371/journal.pone.0294635)
Supplement: S6 File — This file shows additional findings in included studies. (PDF) [file pone.0294635.s006.pdf]

Supplementary File 6. Additional findings in included studies.

Study	Additional Findings
Ali SR *et al.* 2023	*Adequate technology resources were present to meet VMDTM requirements during COVID-19*: yes, 68.1%.
Amin NB *et al.* 2023	*Have you cared for a patient who was discussed during a VMDTM*: yes, 84%; no, 12%; not specified, 4%.<br>*VMDTs allow adjuvant care in a timely manner*: yes, 88%; neutral, 6%; no, 2%; community oncologist vs academic oncologist, 64% vs 40%.<br>*How soon were patients referred/evaluated for adjuvant care since start of VMDTs*: similar, 22%; 1-week sooner, 24%; 2-weeks sooner, 32%; 3-weeks sooner, 8%; not specified, 14%; community vs academic physicians, 64% vs 40%.<br>*VMDTs have allowed for better retention of patients for non-surgical care (n=27 oncologists)*: agree, 70.3% agreed; neutral, 25.9%; disagree, 0%; community oncologist vs academic oncologist, 46% vs 20%.<br><br>No significant difference in responses between facility or specialty type.
Groothuizen JE *et al.* 2023	[D] Opposing views on ability to focus during meetings. Some said that it has improved, while others said it has worsened.<br>[D] Participants do not have to focus during the whole meeting as they can multitask when their contribution is not needed or their participation is not required.
Soukup T *et al.* 2023	*In what way has access to referring cases changed since COVID-19 (n=403)*: 49.19.<br>*Have the invitation list for attendance changed since COVID-19 (n=403)*: 51.53.<br>*Were sufficient IT resources available to make improvements or cope with IT-related changes necessitated by COVID-19 (n=392)*: 41.41<br>*Meeting attendance since COVID-19*: 46.41<br>*Available time to discuss complex cases since COVID-19 (n=392)*: 47.66.<br>*Change in opportunity to present patients known to MDT members since COVID-19 (n=384)*: 49.09.<br>*Change in representation of patients during meetings since COVID-19 (n=384)*: 50.18.<br>*Opportunities to discuss patients who would most benefit from MDT review since COVID-19 (n=375)*: 47.98.<br>*Quality of information presentation since COVID-19 (n=375)*: 51.15.<br>*Participation in meeting discussion since COVID-19 (n=375)*: 50.02.<br><br>No statistically significant differences between seniority levels, cancer specialty, academic or local hospitals.
Thiagarajan S *et al.* 2023	*Reasons for patients sent for discussion in VMDTM*: no consensus at tumor board discussion, 21.2%; second opinion, 3.8%; no clear guidelines, 15.4%; all of the above, 40%; no response, 9.6%.<br>*Implementability of VMDTM decisions*: usually, 75%; always, 17.3%; sometimes, 5.8%; rarely, 1.9%.<br>*Reasons for not implementing VMDTM decisions*: lack of expertise/infrastructure, 28.8%; disagreement, 11%; others, 13.4%.
Bonanno N *et al.* 2023	*Attendance in MDTMs*: mandatory, 127 (62.3%); not mandatory, 77 (37.7%).<br>*COVID-19 and social restrictions led to VMDTMs*: yes, 157 (77%); VMDTMs exist prior to the pandemic, 12 (5.9%); have not implement nor priorly used VMDTMs, 35 (17.2%)<br>*Initial reaction to shift to VMDTMs*: approved the change, 141 (69.1%); neutral towards change, 57 (27.9%); disapproved the change, 6 (3%).<br>*Comparison of pre-pandemic and during-pandemic VMDTM frequency*: same, 144 (70.6%); 44 (21.6%); increased frequency and/or number, 44 (21.6%); decreased frequency and/or number, 16 (7.8%).<br>*Major benefit of VMDTMs*: safer than IMDTMs, 164, (81.4%).<br>*Role of radiologists**: unchanged, 77.9%; changed, 22.3%.<br>*Predicted workload for radiologists if VMDTMs continue in the future*: same, 112 (54.9%); increase, 77 (37.7%); decrease, 15 (7.4%)<br><br>*Meetings are more radiology-driven, radiologists have more control over the discussion, there is increased interaction with other members, radiologists have to deal with technical difficulties more often compared to in-person MDTM.
Esteso F *et al.* 2022	N
Pearlmutter B *et al.* 2022	[C] *Ease of participation in offsite location*: median score, 7.<br>[C] *Ease of adding cases*: median score, 0.<br>*Better video-feeds will improve VMDTMs*: median score, 3; significantly more for surgeons vs others, 5 vs. 2, p=0.03.<br>*Hybrid nature will improve VMDTMs*: median score, 3.<br>*Different interface will improve VMDTMs*: median score, 1.<br>*If social distancing was mandatory, would you prefer a VMDTM*: yes, 63.1%.
Thallinger C et al. 2022	*Reason of participation*: personal motivation, 43%; case-specific questions, 43%; networking, 2%<br>*Motivation to present a case*: general discussion 36%; ICPI side effect management, 29%; ICPI-related treatment options, 14%.<br>*Expectation regarding main motivation was met*: yes, 88%.
Cathcart P *et al.* *2021*	N
Mohahembhai *et al.* 2021	N
Rajasekaran RB *et al.* 2021	*Approved change to VMDTMs at the start of the pandemic*: yes, 72.2%.<br>38.9% felt it significantly affected patient care.<br>*Pandemic affected care*: yes, 100%.<br>*VMDTMs are the future of cancer care*: yes, 77.8%.<br>*Pandemic experience would lead to global MDTMs*: yes, 91.7%.
Rosabal-Obando M *et al.* 2021<br><br>*Approximate value from figure.	*Recommendations were used in similar cases*: agree, 99%*; disagree, 1%*.<br>*Recommendations have been ingrained in internal protocol*: agree, 96.1%*; neutral, 2.9%*; disagree, 1%*.
Dharmarajan H *et al.* 2020	N
Habermann TM *et al.* 2020	N
Pan M. *et al* 2020	N
Rosell L *et al.* 2020	N
Sidpra J *et al.* 2020<br><br>*Approximate value from figure.	[C] *Continuity of care*: equal, 71%*; equal or better, 100%.
Rosell L *et al.* 2019	*Goals of national MDTM are clear**: score 1-3, 17%; score 4, 4%; 5-7, 78%.<br><br>*In agreement with MDT-MOT and MDT-MODe
van Huizen LS *et al.* 2019	N
Abu Arja MH *et al.* 2018	*Post-meeting notes were sent within appropriate time (n=95)*: totally agree, 93%.<br>*Post-meeting notes were easy to understand (n=95)*: totally agree, 92%.<br>*More sub-specialties are needed (n=84)*: totally agree, 86%.<br>*Reviewed second opinion of pathology (n=84)*: yes, 57%; rarely/never, 31%.
Crispen C *et al.* 2014	*Facilitates self-regulation of radiation oncology practice*: agree, 100%; mean score, 3.3 ± 0.5.
Marshall CL *et al.* 2014	*Discussion was relevant to practice*, 4.6 ± 0.6.
Shea CM *et al.* 2014	*Current program can accommodate more cases*: yes, 72%; no, 3%.

Bold RJ *et al.* 2013	N
Stevenson MM *et al.* 2013	N
Schroeder JK et al. 2011	*Expectations from the VMDTM*: receive expert advice, 81.32%; quality improvement for the patient, 73.63%; generate second opinion, 62.64%; advanced educational training, 58.24%; cooperation with colleagues from other sectors, 39.56%; saving time, 23.08%; other, 1.09%.
Chekerov R *et al.* 2008	N
Kunkler IH *et al.* 2007	N
Savage SA *et al.* 2006	*Minutes of the meeting should be used for basis for*: formal review of specific case man, 94%; an annual 'lessons learned' document, 88%; development of a teaching aid for trainees, 68%.
Kunkler I *et al.* 2006	N
Delaney G *et al.* 2004	*Will be more likely to attend a VMDTM if there was a Medicare rebate*: yes, 100%.
Gagliardi *et al.* 2003	*Topic was relevant to practice*: agree, 74.6%; neutral, 17.9%; disagree, 4.5%; no response, 3.0%.<br>*Preferred frequency of meetings*: weekly, 50%; monthly, 31.3%; fortnightly, 13%; I will not attend, 6.3%.
Oliver IN *et al.* 2000	N
Hunter *et al.* 1999	*Lighting quality*: excellent/good, 97%.
	Lighting quality: excellent/good, 100%.

Abbreviations: ICPI, immune checkpoint inhibitors; IT, information and technology; MDT-MODe, multidisciplinary team-metric of decision-making; MDT-MOT, multidisciplinary team-meeting observational tool; MDTM, multidisciplinary team meeting; N, none; VMDTM, virtual multidisciplinary team meeting; COVID-19, coronavirus disease of 2019.

[C] Indicates that the finding compares VMDTM with IMDTMs. Where numbers are quoted, VMDTMs have been compared with IMDTMs i.e. VMDTM vs. IMDTM.

[D] Indicates that the finding was qualitatively cited, either through the use of an interview, free-text response, an anthropological analysis (Delaney *et al.*), or as a summary from the study's data which was not otherwise quantitatively stated in the relevant paper.
