# Supplementary material for: How well does the virtual format of oncology multidisciplinary team meetings work? An assessment of participants’ perspectives and limitations: A scoping review
Source: PLoS One. 2023 Nov 16;18(11):e0294635. doi: 10.1371/journal.pone.0294635 (PMC10653537; doi:10.1371/journal.pone.0294635)
Supplement: Attachment — Submitted filename: Response to Reviewers.docx [file pone.0294635.s007.docx]

**Rebuttal Letter**

**Manuscript ID**	PONE-D-23-30802

**Title**	How well does the virtual format of oncology multidisciplinary team meetings work?	An assessment of participants’ perspectives and limitations: a scoping review

I would like to thank both reviewers for taking the time out to read our manuscript and for their interest and their comments.

Reviewer 1 had no comments for our paper. We have addressed all the comments raised by Reviewer 2.

Comment:	Line 47 - you set up VMDTM as a "promising solution" however, you hadn't	introduced a problem yet.

Response:	The sentence has been edited to highlight that the solution is for geographical barriers,	as was conveyed by the latter half of the sentence. I thank the reviewer for raising	this comment.

Comment:	Line 60 - define IMDTMs before use.

Response:	Thank you for this comment. The abbreviation has been defined.

Comment:	Line 62 - this comment likely falls outside of the context of your scoping review;	however, I'm curious if there were perception changes pre-during-post pandemic…

Response:	This is an interesting comment. I agree with the reviewer that evaluating a change in	perception about VMDTMs before and after the COVID pandemic, is actually not	the focus of this study and it also does not sit well with the nature of this review. It	would perhaps form the basis for a new systematic review which specifically focuses	on this particular question.

Despite this, I would like to point out that in accordance with the scoping nature of	our review, we have, indeed made the distinction for studies that were published	during or after the COVID-19 pandemic (*n*=10). This has been stated in Table 1 and	the results section (Line 140),

Comment:	Line 274 contradicts 278. VMDTMs are good to meet other specialities and then it	says networking with other specialities is difficult and relationships deteriorate. In the	attendence section (165) you did a better job with transition... some reports state its	good, however, others its bad. Use a transition in the paragraph, please.

Response:	I would like to respond to this comment by highlighting the sentences in the	manuscript. We state that “*VMDTM are good to meet other specialties*…”, which is	followed by “*However, maintaining these working relationships with members is	difficult*”. Although VMDTMs help physicians meet physicians from other	specialties, it does not translate into long-lasting professional relationships which is	why “*When compared to IMDTMs, networking with other specialties is	difficult and interpersonal	relationships deteriorate”.*

I welcome the reviewer’s comment and the opportunity to respond. But as stated by	the quotations above, there are two things to consider: (1) That the distinction	between “meeting” and “maintaining relationships” is to be considered, which is	conveyed by the current text; (2) that the second sentence is actually comparing	VMDTMs to IMDTMs, and is not about VMDTMs entirely.

Comment:	Line 382 - first overall review or first scoping review?
Response:	This is the first overall review that assesses VMDTM feasibility through the eyes of	VMDTM participants. I have edited the sentence to highlight the distinction as	recommended by the reviewer.

Comment:	Line 435 - "this allows this" awkward

Response:	We have restructured the sentence from “*This allows this paper to form the basis…*”	to “*Therefore, our study forms the basis…*”

Comment:	PRISMA Flow:make records identified from Databases N=2617 (the individual	databases can be "little n") I have slight concern that you distrinction of new studies	vs reports of new included studies incorrectly. seems like the new studies are from	the "left side" and the reports are from the "right side" It could be correct, but it's a	big coincidence when looking at left vs. right. Check this out, it's the best PRISMA	clarifications that I've come across: DOI 10.5195/jmla.2022.1449
Response:	I thank you for this particular comment, and will elaborate both aspects of the	question. The lowercase “n” is used to denote the number of studies throughout the	flowchart. And so, it denotes the number of studies at “Databases n=2617” and not	the number of databases. Since the essence of the variable n has not changed, it	seems best to quote it as such. I agree with the reviewer that an uppercase “N” should	be used,	only if N represented something else than n. The lowercase n in the figure is	also in-line with the official PRISMA 2020 flowchart which can be found here	(http://prisma-statement.org/prismastatement/flowdiagram.aspx). Similarly, the	distinction of new included studies and the reports of new included studies is also in-	line with the PRISMA 2020 flowchart. We kept our citation search in the second	column because it was conducted as a secondary search. This is in-line with the	article quoted by the reviewer - which was indeed an interesting read.

Comment:	Supplement 4 - the final row (breast VMDTM) is missing author information.
Response:	The final row belongs to the study by Hunter et al. Therefore, I have merged the	cells to indicate as such.

Comment:	Supplement 6 - the final row (Lighting quality 100%) is missing author information.

Response:	The final row belongs to the study by Hunter et al. Therefore, I have merged the	cells to indicate as such.
